# Resting-State Magnetoencephalography Functional Connectivity in Cervical Spondylotic Myelopathy: An MEG Study with SHAP-Based Interpretation

**DOI:** 10.3390/bioengineering13090988

**Published:** 2026-08-27

**Authors:** Geng Zhao, Zhuang Miao, Shiqiang Zheng, Xinyu Liu, Xu Zhang

**Affiliations:** 1 School of Instrumentation and Optoelectronic Engineering, Beihang University, Beijing 100191, China; by2243211@buaa.edu.cn (G.Z.); by2243212@buaa.edu.cn (Z.M.); zhengshiqiang@buaa.edu.cn (S.Z.); 2 Hangzhou National Institute of Extremely-Weak Magnetic Field Infrastructure, Hangzhou 310051, China; 3 Cheeloo College of Medicine, Shandong University, Jinan 250061, China

**Keywords:** MEG, CSM, functional connectivity, machine learning, SHAP

## Abstract

The diagnosis of cervical spondylotic myelopathy (CSM) relies mainly on clinical symptoms and structural imaging, highlighting the need for objective functional biomarkers. This study investigated alterations in resting-state magnetoencephalography (MEG) functional connectivity in CSM and evaluated whether multiband weighted phase lag index (wPLI) features could distinguish CSM patients from healthy controls (HCs). Eyes-closed resting-state MEG data were acquired from 31 CSM patients and 32 HCs. Region-of-interest-level wPLI connectivity was calculated in the theta, alpha, beta, and gamma bands and used to train multiple machine learning classifiers. Model performance was assessed using nested group cross-validation, and SHapley Additive exPlanations (SHAP) were used to interpret the best-performing model. Patients with CSM exhibited frequency-specific connectivity alterations, particularly in the theta and gamma bands. Logistic regression achieved the best overall discriminative performance, and SHAP analysis indicated that classification was driven mainly by long-range theta-band connections and gamma-band connections involving the frontal pole. These findings suggest that CSM is associated with measurable reorganization of large-scale cortical networks and that resting-state MEG connectivity combined with explainable machine learning may provide a promising framework for exploring candidate neurophysiological biomarkers of CSM.

## 1. Introduction

Cervical spondylotic myelopathy (CSM) is a progressive neurological disorder caused by compression of the spinal cord at one or more cervical levels [1]. Its major clinical manifestations include: (1) motor dysfunction, such as reduced lower-limb muscle strength and gait disturbance; (2) sensory abnormalities, including limb numbness and a girdle-like sensation in the trunk; and (3) autonomic dysfunction, particularly bladder and bowel impairment [2,3]. Surgical decompression, performed via anterior or posterior approaches, remains the mainstay of treatment for CSM. However, public awareness of cervical spondylosis is limited [4], and the early symptoms of CSM are often subtle and difficult to recognize, which may delay medical consultation until the disease has progressed beyond the optimal therapeutic window [5]. In general, longer symptom duration and greater disease severity are associated with poorer postoperative outcomes [6,7]. Conventional diagnosis of CSM primarily relies on clinical symptoms and structural imaging findings. Nevertheless, imaging mainly reflects anatomical changes, whereas functional assessment can be influenced by subjective factors. Therefore, there is a clear need for more objective biomarkers that can better characterize the functional status of CSM patients.

Resting-state magnetoencephalography (MEG), with its millisecond temporal resolution and high sensitivity to neural oscillatory activity, provides a unique noninvasive approach for investigating the dynamic functional organization of brain networks. Spontaneous neural activity recorded during rest can reliably reflect intrinsic network architecture associated with underlying pathophysiological processes. Functional connectivity analysis enables assessment of synchronization between spatially distributed brain regions, which is particularly relevant to CSM because neurological dysfunction may arise not only from focal cervical cord compression but also from broader reorganization of sensory, motor, and higher-order integrative or compensatory networks. Among available functional connectivity metrics, the weighted phase lag index (wPLI) is particularly advantageous because of its relative robustness against zero-phase-lag coupling induced by field spread or volume conduction. Compared with metrics such as phase-locking value (PLV) and coherence, wPLI reduces the influence of interactions that may be spuriously driven by volume conduction [8,9,10]. Accordingly, wPLI is well suited to detecting time-lagged functional coupling between neural signals [11,12], while minimizing spurious zero-lag associations arising from mixed true and artifactual relationships [13]. On this basis, the present study used wPLI to construct high-dimensional functional connectivity networks across multiple regions of interest (ROI) and canonical frequency bands, with the aim of comprehensively characterizing potential spectral and spatially specific network abnormalities in CSM.

Machine learning provides a powerful analytical framework for modeling complex, high-dimensional, and multifactorial data, often outperforming conventional statistical approaches in such settings [14]. In recent years, machine learning methods have increasingly been applied to identify diagnostic and prognostic factors in spinal disorders using heterogeneous data sources that are difficult to evaluate directly with conventional approaches. For example, Koyama et al. proposed a screening model for degenerative cervical myelopathy based on video recordings of grasping movements [15,16], while Hopkins et al. developed an algorithmic model for the early diagnosis of CSM, particularly in cases in which symptoms and imaging abnormalities are not yet prominent [17]. Because wPLI-derived connectivity features are intrinsically high-dimensional across multiple frequency bands and ROIs, conventional univariate and even standard multivariate analyses may be insufficient to capture the relevant combinatorial patterns. At the same time, the “black-box” nature of machine learning models may limit the biological interpretability of their outputs. To address this issue, the present study incorporates explainable artificial intelligence techniques, specifically SHapley Additive exPlanations (SHAP). This framework enables both model development and identification of the functional connections and frequency bands that contribute most strongly to distinguishing CSM patients from healthy controls (HCs).

Based on the above considerations, this exploratory study was designed to test the central hypothesis that patients with CSM exhibit specific alterations in resting-state MEG functional connectivity that may serve as objective neurophysiological biomarkers. The specific aims of the study were as follows: (1) to construct machine learning models based on multiband wPLI connectivity features for discriminating CSM patients from HCs; (2) use SHAP to interpret model outputs and identify the connections and frequency bands that contribute most strongly to classification; and (3) to examine associations between these key network features and clinical scale scores to assess their biological interpretability and potential clinical relevance.

## 2. Materials and Methods

### 2.1. Design and Patient Population

CSM patients were recruited from the Spine Surgery Ward of Qilu Hospital, Shandong University, between January 2024 and July 2024. Additionally, healthy volunteers were recruited from the community. The inclusion criteria for CSM patients were: (1) age between 18 and 70 years; (2) diagnosed with CSM; (3) imaging evidence of spinal cord compression. Exclusion criteria included: (1) history of spinal surgery; (2) diagnosis of other types of cervical spondylosis; (3) presence of other neurological or systemic diseases; (4) presence of pacemakers or other metal implants in the body. This study was approved by the Ethics Committee of Qilu Hospital (Approval No. KYLL-202312-015-1). All participants were fully informed about the study details and provided written informed consent.

### 2.2. Clinical Assessments

In this study, the Japanese Orthopaedic Association (JOA) score was used to assess patients’ functional status, a widely recognized method for evaluating the severity of neurological symptoms in cervical spondylosis patients [18]. Pain intensity was assessed using the Visual Analog Scale (VAS), and quality of life was evaluated using the 36-Item Short Form Health Survey (SF-36) [19]. The eight SF-36 domains were Physical Functioning (PF), Role Physical (RP), Bodily Pain (BP), General Health (GH), Vitality (VT), Social Functioning (SF), Role Emotional (RE), and Mental Health (MH). Using a commonly adopted rule-based approach, two composite indices were constructed: a physical composite derived from PF, RP, BP, and GH, and a mental composite derived from VT, SF, RE, and MH. To preserve interpretability and maintain scale consistency, both composite scores were calculated as equal-weight averages of the corresponding four 0–100 domain scores, resulting in composite scores also ranging from 0 to 100.

### 2.3. MEG Acquisition

**Figure 1:**
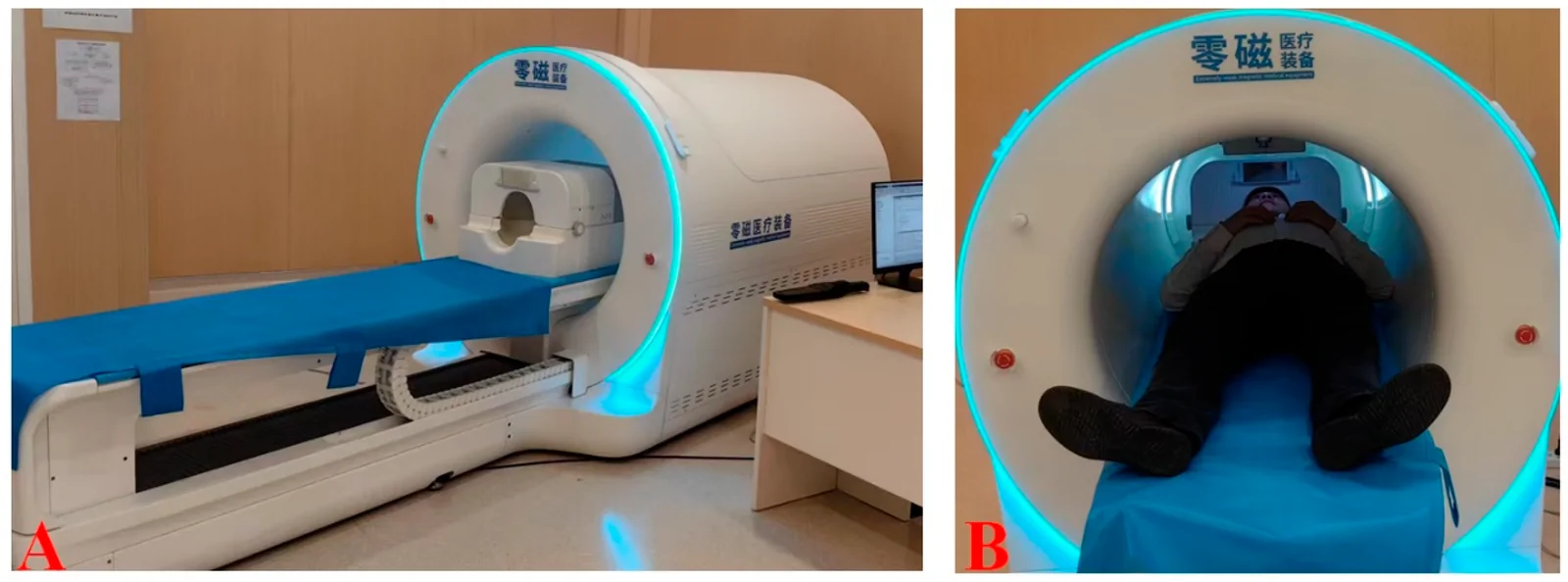
Side view (**A**) and front view (**B**) of the MEG device.

Resting-state MEG recordings were conducted with participants in an eyes-closed state (Figure 1). Participants were instructed to lie comfortably on the examination bed, gently close their eyes, relax, avoid deliberate mental activity, and remain awake throughout the recording. Before formal data collection, participants were familiarized with the procedure to minimize anxiety and unnecessary movement. Quality control was maintained throughout the recording, and continuous video monitoring using a camera integrated into the MEG system was used to confirm wakefulness and ensure participant safety. A recording duration of 2 min was selected to balance data quality and participant tolerance. Given the clinical characteristics of CSM patients, shorter acquisitions reduce discomfort and motion-related artifacts.

All MEG recordings were performed in a magnetically shielded room (MSR) with a residual magnetic field of less than 10 nT in the central area. All electronic equipment for recording was placed outside the MSR. Brain magnetic data were acquired using a 64-channel brain magnetic array. The MEG sensor locations were defined according to the International 10–20 system. The sensitivity of the single-axis OPM magnetometers (Hangzhou Institute of Extremely-Weak Magnetic Field Major National Science and Technology Infrastructure, Hangzhou, China) in the array was 20 $fT/\sqrt{Hz}$ [20]. Continuous acquisition mode was used, sampling brain magnetic signals at a rate of 1000 Hz.

### 2.4. Preprocessing

Preprocessing was performed offline using standard procedures. Continuous MEG signals were band-pass filtered from 2 to 40 Hz, and notch filters at 27 Hz and 30 Hz were applied to suppress electrical line noise. Following visual inspection of the raw data, segments heavily contaminated by motion artifacts, muscle bursts, or sensor failures were manually rejected. Independent Component Analysis (ICA) was used to identify ocular and cardiac artifacts, which were removed based on their spatial topography and time course. The remaining data were segmented into fixed-length epochs of 5 s duration. Epochs overlapping with any rejected segments were discarded, retaining only artifact-free epochs. Following preprocessing, each participant contributed approximately 10–20 epochs of artifact-free data.

### 2.5. Feature Construction for Machine Learning

Resting-state functional connectivity was quantified using the wPLI, whose core idea is to characterize stable phase-lag relationships using the asymmetry of the phase difference distribution between two signals, thereby mitigating the influence of zero-phase coupling potentially caused by volume conduction/common sources to some extent. For each participant, ROI-level wPLI matrices were calculated within four canonical frequency bands: theta (4–8 Hz), alpha (8–13 Hz), beta (13–30 Hz), and gamma (30–40 Hz). The 64 MEG sensors were grouped into eight sensor-level regions of interest (ROIs) according to their anatomical and topographical locations on the scalp: Occipital, Parietal, Central, Centroparietal, Frontal, Frontal Pole, Temporal, and Frontocentral. The sensors included in each ROI are shown in Supplementary Table S1.

The wPLI was first estimated for individual sensor pairs within each frequency band. For each pair of ROIs, wPLI values for all cross-ROI sensor pairs were subsequently averaged to obtain an ROI-level connectivity estimate. The self-connections were excluded. Because eight ROIs yield 28 unique pairwise connections, 28 connectivity features were generated for each frequency band. Across the four frequency bands, this resulted in a total of 112 candidate connectivity features per participant. These variables constituted the initial feature set for subsequent machine learning analysis.

### 2.6. Multi-Model Selection and Hyperparameter Optimization

Seven supervised classification algorithms were evaluated: logistic regression (LR), support vector machine (SVM), random forest (RF), decision tree (DT), k-nearest neighbors (KNN), Gaussian naive Bayes (NB), and extreme gradient boosting (XGBoost). Missing feature values, if present, were imputed using the median estimated from the corresponding training data. Standardization was additionally applied to LR, SVM, KNN, and Gaussian NB.

To reduce dimensionality and limit overfitting, feature selection was incorporated directly into each machine learning pipeline using SelectKBest with the ANOVA F-statistic. Starting from the 112 candidate connectivity features, the number of retained features (k) was treated as a tunable hyperparameter with candidate values of 10, 20, 30, and 40. Model development and evaluation were performed using a nested group-aware cross-validation framework. The outer loop consisted of five folds and was used exclusively to estimate model generalization performance. Within each outer training set, a four-fold group-aware inner cross-validation was used to jointly optimize the number of selected features and model-specific hyperparameters through grid search. Subject identifiers were used as grouping variables to ensure that data from the same participant could not appear simultaneously in the training and test sets. All preprocessing operations required for model fitting were fitted using only the corresponding training data, minimizing information leakage. The complete specific hyperparameter search spaces are provided in Supplementary Table S2.

For each outer fold, the model configuration yielding the highest mean inner-cross-validation ROC-AUC was refitted using the corresponding outer training set and evaluated on the held-out outer test set. Model performance was evaluated using ROC-AUC, area under the precision-recall (AUC-PR) curve, accuracy, sensitivity, specificity, balanced accuracy, and F1-score. The F1-score corresponds to an F-beta score with β = 1. Model performance was summarized as the mean and standard deviation across the five outer folds. After comparison of all candidate classifiers, the model with the highest mean outer-cross-validation ROC-AUC was selected as the best-performing model. The selected model was subsequently refitted on the complete dataset for final feature interpretation.

### 2.7. Feature Stability Analysis

To assess the reproducibility of feature selection, the features retained by SelectKBest were recorded separately for each outer training fold of the nested cross-validation procedure. For each feature, selection frequency was calculated as the proportion of outer folds in which the feature was retained. Features selected in at least 80% of the five outer folds were considered stable. Mean feature-selection ranks across folds were also calculated. In addition, the similarity of the selected feature sets between pairs of outer folds was quantified using the Jaccard index, defined as the size of the intersection divided by the size of the union of the two feature sets.

### 2.8. SHAP Analysis

To interpret the contribution of key features to the classification of CSM versus HC by the machine learning model, this study employed Shapley Additive exPlanations (SHAP) for explainability analysis after completing nested cross-validation and identifying the best-performing model. The background and explanation samples were randomly drawn from the available transformed observations using a fixed random seed. SHAP values were subsequently summarized using the mean absolute SHAP value to quantify global feature importance, and the highest-ranking features were visualized using summary and dependence plots.

### 2.9. Statistical Analysis

Group differences in MEG resting-state functional connectivity and network topology metrics were assessed by comparing wPLI value matrices. Independent-samples t-tests were used for paired samples, with a two-sided α = 0.05. To address the multiple comparisons problem, the False Discovery Rate (FDR) correction method was applied. Effect sizes for group differences were quantified using Cohen’s d. Connectivity calculations and preprocessing were performed in Python using MNE-Python V3.11 and MNE-connectivity V0.7.0. All stochastic processes used a fixed seed to enhance reproducibility.

## 3. Results

### 3.1. Participants and Clinical Characteristics

Table 1 presents the clinical characteristics of the entire dataset. After MEG data quality assessment and exclusion of recordings with excessive motion artifacts, 31 CSM patients (14 males and 17 females; mean age 58.02 ± 8.01 years) and 32 healthy volunteers (15 males and 17 females; mean age 58.32 ± 7.53 years) were included. The mean VAS score among patients was 3.32 ± 1.64, and the mean JOA score was 11.33 ± 1.72. No significant group differences in age or sex were observed.

### 3.2. Group Differences in wPLI Connectivity

**Figure 2:**
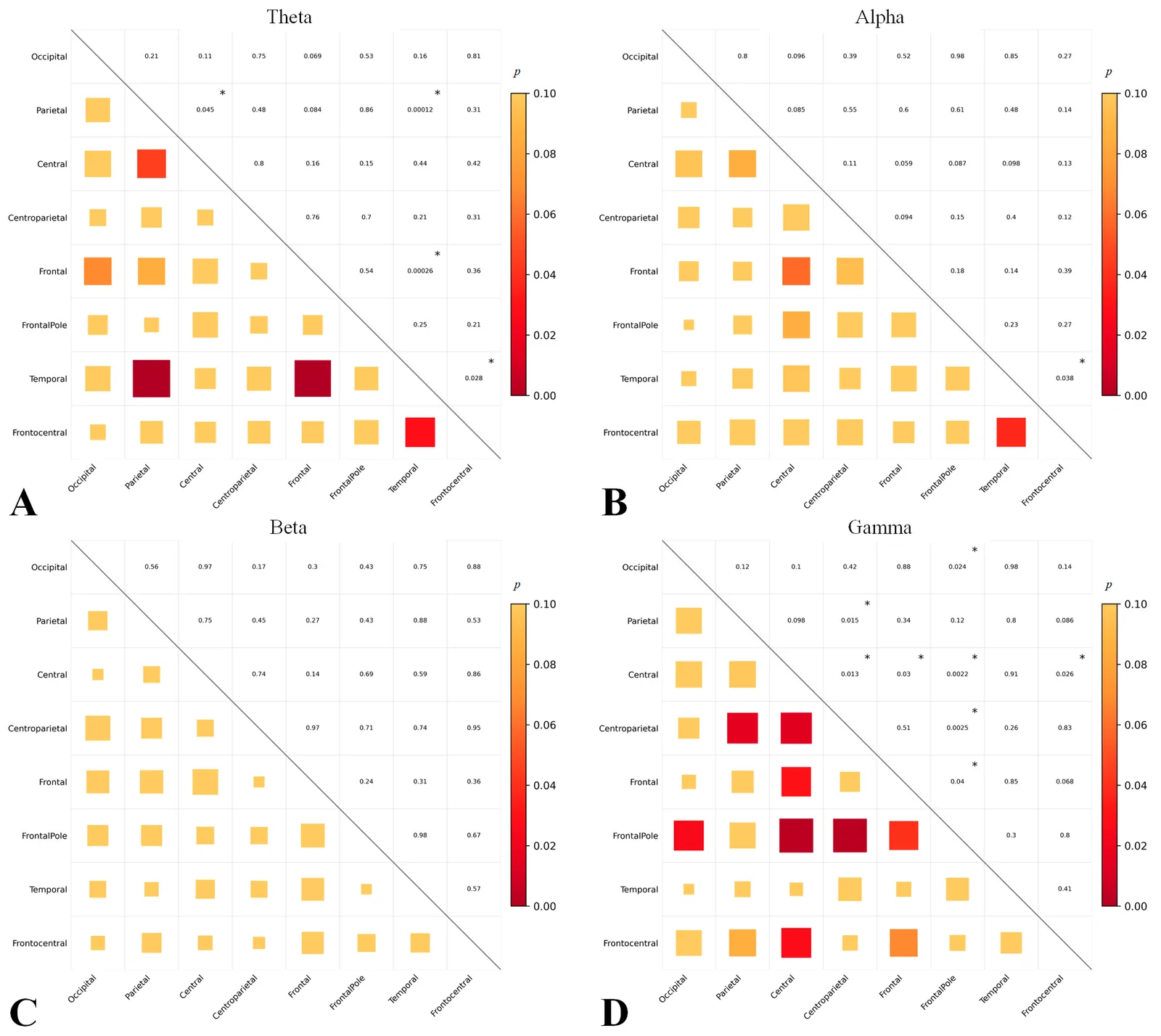
*p*-values for wPLI between the CSM and HC groups in the theta (**A**), alpha (**B**), beta (**C**), and gamma (**D**) frequency bands. * *p* < 0.05.

Figure 2 displays the *p*-value matrices for wPLI differences between the HC and CSM groups in the theta, alpha, beta, and gamma frequency bands. In the uncorrected analyses, significant wPLI differences (*p* < 0.05) were found for the Parietal-Temporal, Frontal-Temporal, Temporal-Frontocentral, and Parietal-Central connections in the theta band and the Temporal-Frontocentral connection in the alpha band; and the Central-Frontal Pole, Centroparietal-Frontal Pole, Central-Centroparietal, Parietal-Centroparietal, Occipital-Frontal Pole, Central-Frontocentral, Central-Frontal, and Frontal-Frontal Pole connections in the gamma band. After FDR correction, significant differences remained for the theta-band Parietal-Temporal (p_adj_ = 0.014, Cohen’s d = 1.06) and Frontal-Temporal (p_adj_ = 0.030, Cohen’s d = 0.98) connections.

Among CSM patients, associations between the key connectivity features and clinical measures, including JOA, VAS, PCS, and MCS, were examined. In total, 10 key features were tested against four clinical scales using two correlation methods. In the uncorrected analysis, Gamma-Parietal-Centroparietal connectivity showed a trend toward a negative correlation with MCS (r = −0.362, *p* = 0.045). However, this association did not remain statistically significant after correction for multiple comparisons.

### 3.3. Development and Validation of Machine Learning Models

**Figure 3:**
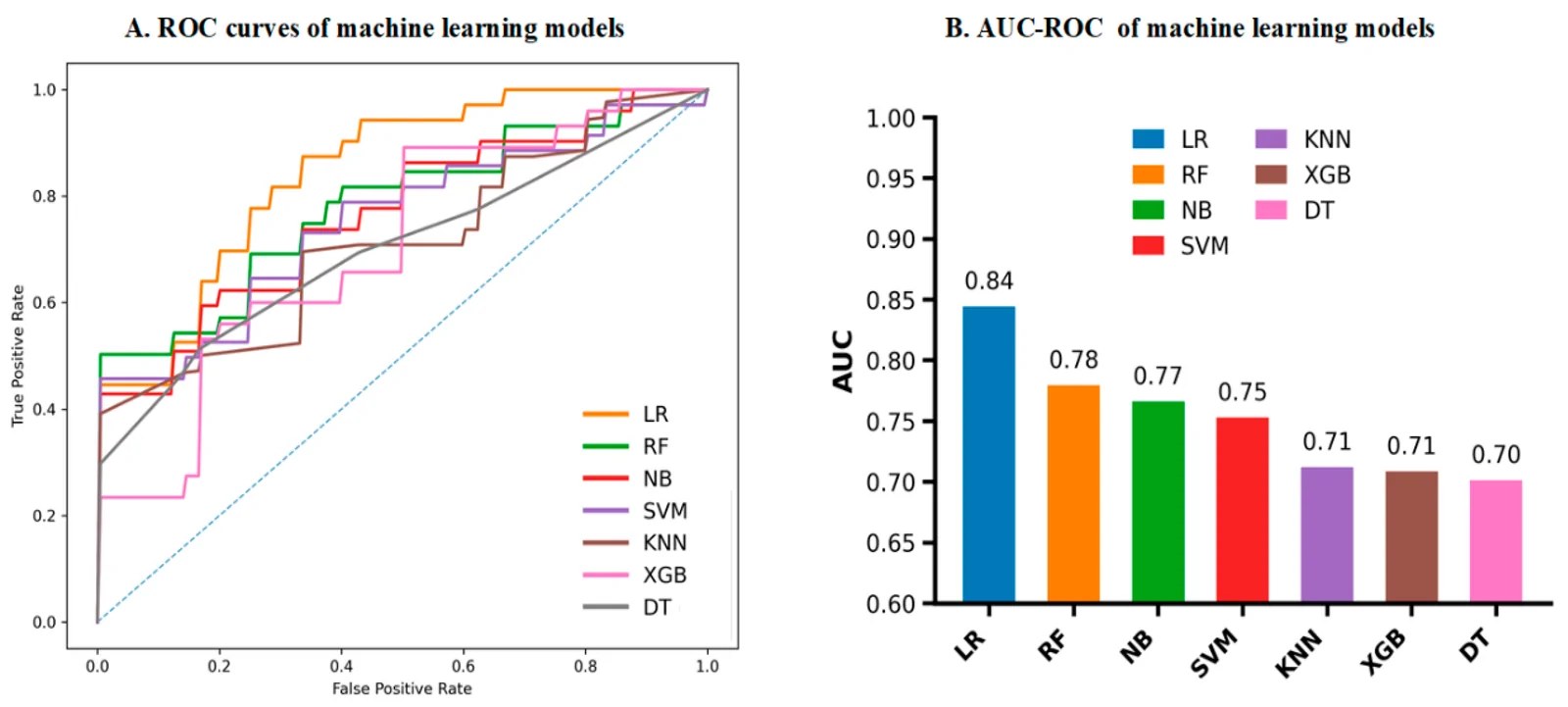
The predictive performance of machine learning approaches. (**A**) ROC curves for the machine learning models. (**B**) Bar plot of AUC values for the machine learning models. ROC, receiver operating characteristic; AUC, area under the curve; LR, logistic regression; RF, random forest; NB, naive Bayes; SVM, support vector machine; KNN, k-nearest neighbors; XGB, extreme gradient boosting; DT, decision tree.

We employed nested subject-wise cross-validation with group separation and divided the data into five folds. In each outer iteration, 4/5 of the data constituted the outer training set and the remaining 1/5 served as the held-out test set. Within each outer training set, four-fold inner group-aware cross-validation was used for feature selection and hyperparameter optimization. Multiple classifiers were evaluated using ROI-pair features derived from wPLI across the theta, alpha, beta, and gamma bands. Inner cross-validation was performed to select model hyperparameters and assess the discriminative ability of seven classifiers for distinguishing CSM from HC. Table 2 presents the performance of the seven machine learning models in terms of sensitivity, specificity, F1-score, accuracy, area under the receiver operating characteristic (ROC) curve, and area under the precision-recall curve (AUC-PR). The ROC curves of the models are shown in Figure 3.

Overall, LR performed best on the two core discriminative metrics, with a mean outer-fold AUC of 0.844 ± 0.027 and AUC-PR of 0.854 ± 0.050. In the present dataset, these results indicated that LR provided the most stable overall discriminative performance. RF achieved the next-highest AUC (0.780 ± 0.046) but showed greater variability in AUC-PR across folds (0.812 ± 0.119). SVM achieved a moderate AUC (0.753 ± 0.107) and a relatively stable AUC-PR (0.816 ± 0.040). In contrast, DT and XGB showed larger standard deviations across folds, indicating less stable generalization across the outer test folds in this study.

### 3.4. Importance of Features Interpreted by SHAP Value

**Figure 4:**
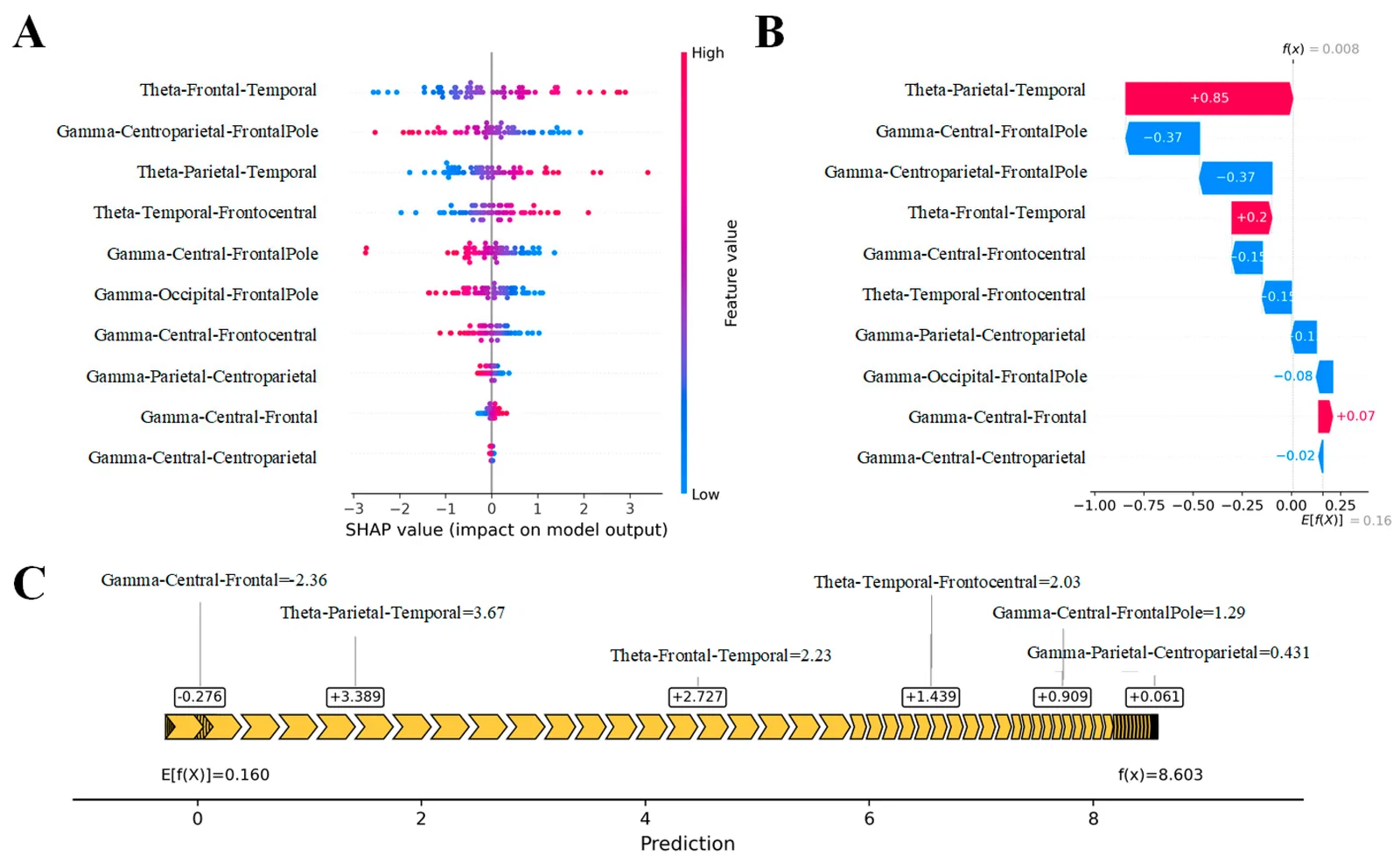
SHAP values for the logistic regression model. (**A**) SHAP summary plot. (**B**) SHAP waterfall plot. (**C**) SHAP force plot. SHAP, SHapley Additive exPlanations.

To clarify the basis of discrimination by the best-performing model (LR), SHAP was used to interpret model output (Figure 4). As LR was identified as the best-performing classifier, the SHAP results reported in the main analysis were obtained using LinearExplainer, which is specifically designed for linear models. At the global level, the SHAP summary plot showed that model discrimination was primarily driven by a small number of connectivity features. The highest-contributing features were concentrated in two patterns: (1) long-range cortical connections in the theta band, including Theta-Frontal-Temporal, Theta-Parietal-Temporal, and Theta-Temporal-Frontocentral connectivity; and (2) frontal-pole-related connections in the gamma band, including Gamma-Centroparietal-Frontal Pole, Gamma-Central-Frontal Pole, and Gamma-Occipital-Frontal Pole connectivity. In this model, these theta- and gamma-band connections contributed strongly to classification decisions.

Regarding the direction of feature effects, higher theta-band Frontal-Temporal and Parietal-Temporal connectivity values were more often associated with positive SHAP values and therefore shifted model output toward the positive class (CSM). In contrast, higher values of several gamma-band frontal-pole-related connections were more often associated with negative SHAP values and therefore shifted model output away from the positive class.

Feature-stability analysis of the LR model identified 12 connectivity features that were selected in at least 80% of the outer cross-validation folds. Seven features were consistently selected in all five folds, including Theta-Parietal-Temporal, Theta-Frontal-Temporal, Gamma-Centroparietal-Frontal Pole, Gamma-Central-Frontal Pole, Gamma-Parietal-Centroparietal, Gamma-Central-Frontocentral, and Alpha-Temporal-Frontocentral. Theta-Parietal-Temporal and Theta-Frontal-Temporal showed particularly high stability. The mean pairwise Jaccard similarity of the complete selected feature sets was 0.364, suggesting variability in the overall feature composition across folds but the presence of a reproducible core subset. Consistent with this finding, Theta-Frontal-Temporal, Theta-Parietal-Temporal, and Gamma-Centroparietal-Frontal Pole were selected in all five outer folds and were also the high-ranking features in the SHAP analysis.

## 4. Discussion

This study investigated brain network abnormalities and their discriminative potential in patients with CSM by integrating resting-state MEG-derived multiband ROI-level wPLI connectivity features with machine learning and SHAP-based model interpretation. Three principal findings emerged. First, compared with healthy controls, CSM patients exhibited altered functional connectivity in a subset of interregional couplings, with the most robust abnormalities observed in the theta band. Second, among the tested classifiers, logistic regression demonstrated the best overall discriminative performance in the present sample, indicating that disease-related information was captured by a relatively compact set of connectivity features, including a reproducible core subset across outer folds. Third, SHAP analysis showed that the model output was primarily driven by a limited number of features, particularly long-range theta-band connections involving frontal, parietal, temporal, and frontocentral regions, as well as gamma-band connections associated with the frontal pole. Taken together, these findings suggest that CSM may be associated with frequency-specific large-scale network reorganization that can be detected by resting-state MEG and incorporated into an explainable classification framework.

In the group comparison, CSM patients exhibited abnormal resting-state functional connectivity across multiple frequency bands, although the most prominent alterations were concentrated in the theta and gamma bands. This pattern suggests that the neural effects associated with CSM may be frequency-dependent rather than uniformly distributed across oscillatory bands. Theta-band oscillations are generally associated with large-scale information integration, cognitive control, sustained attention, and coordinated processing of sensory input [21,22,23]. Previous neuroimaging and electrophysiological studies have suggested that cross-regional synchronization in low-frequency oscillations often undergoes compensatory reorganization under conditions of chronic afferent disturbance, motor pathway injury, or sensorimotor mismatch [24,25,26,27]. Patients with CSM experience prolonged spinal cord compression, under which both ascending sensory input from the periphery and spinal cord and descending motor commands may be persistently disrupted. Accordingly, the theta-band connectivity alterations observed among frontal, parietal, temporal, and frontocentral regions may reflect compensatory network adaptations aimed at maintaining postural control, gait regulation, sensory integration, and cognitive monitoring. In particular, the significant alterations observed in parietal-temporal and frontal-temporal connectivity suggest that the central effects of CSM may extend beyond the classical primary sensorimotor network to association cortices involved in spatial attention, sensory integration, and higher-order information processing.

In addition to the theta-band abnormalities, model interpretation highlighted gamma-band frontal-pole-related connections as important contributors to classification. In the present study, gamma-band differences were mainly observed in connections between the frontal pole and the central, centroparietal, occipital, and frontocentral regions. Gamma oscillations are typically linked to rapid synchronization of local neuronal assemblies, fine-grained sensory processing, task-related resource allocation, and higher-order executive regulation [28]. As the most anterior part of the prefrontal cortex, the frontal pole has been implicated in complex behavioral selection, maintenance of delayed intentions, monitoring of internal representations, and integration of information across higher-order control networks [29,30]. Several frontal-pole-related gamma-band connections showed prominent uncorrected between-group differences and also ranked highly in the SHAP analysis. One possible explanation is that chronic spinal cord compression reduces the quality of sensory afferent input and impairs motor output efficiency, thereby requiring greater engagement of prefrontal systems in the higher-order integration of posture, motor planning, and sensory feedback; this process may be reflected in altered frontal-pole-related high-frequency connectivity. Alternatively, these gamma-band changes may reflect a persistent compensatory load, with relevant cortical circuits remaining abnormally synchronized even in the absence of an explicit task. Although the precise mechanism remains uncertain, the present findings suggest that CSM-related brain network abnormalities may involve both low-frequency large-scale coupling and high-frequency long interactions.

The univariate statistical analysis and machine learning interpretability results showed relatively good consistency. After FDR correction, theta-band parietal-temporal and frontal-temporal connections remained significant, and both also ranked among the top SHAP features. This convergence suggests that at least part of the discriminative information captured by the model was associated with connectivity features that also showed robust between-group differences. It also indicates that machine learning may identify multifeature patterns with moderate effects that are not fully captured by conventional univariate testing. The superior performance of logistic regression over several nonlinear models in this sample may further suggest that the key differences between CSM and HC were represented primarily by relatively stable linear combinations of connectivity features rather than by highly complex nonlinear decision boundaries.

The present findings may also have clinical relevance. Current diagnosis of CSM relies primarily on neurological examination and structural imaging, yet the severity of spinal cord compression on imaging does not always fully explain functional impairment, and symptom-based evaluation may be influenced by subjectivity. Resting-state MEG connectivity may therefore offer complementary information by capturing the functional consequences of cord compression at the cortical network level. In this regard, multiband wPLI features may serve as candidate objective biomarkers of disease-related neurophysiological reorganization. The present findings should be interpreted as evidence of methodological feasibility rather than clinical diagnostic validation. Although the nested cross-validation framework provided an internally validated estimate of discriminative performance while maintaining subject-level independence, the relatively small single-center sample does not permit definitive conclusions regarding clinical diagnostic accuracy or generalizability. Larger multicenter studies with independent external validation will therefore be necessary to determine the reproducibility, diagnostic utility, and clinical applicability of these candidate MEG connectivity biomarkers.

Several limitations of this study should be acknowledged. First, the sample size was relatively limited. Although nested grouped cross-validation was employed to obtain an estimate of generalization performance with minimal bias, model performance and feature importance may still have been influenced by interindividual variability in this moderate-sized sample. Although nested group cross-validation was used to reduce overfitting and obtain an unbiased estimate of model performance, independent external validation remains necessary. Future multicenter studies with independent cohorts are required to determine the reproducibility, robustness, and clinical applicability of the identified connectivity biomarkers. Second, this study used sensor-level ROI parcellation rather than connectivity analysis based on source-space reconstruction of cortical or deep structures. Although wPLI is relatively robust to volume conduction, sensor-level connectivity may still be affected by spatial mixing. Future work could therefore incorporate source localization techniques to derive network maps that more closely reflect the underlying neural generators. Third, the present study was cross-sectional and therefore could not determine whether the observed brain network abnormalities represented consequences of disease progression, compensatory mechanisms, or a combination of both. Longitudinal follow-up, particularly perioperative assessment before and after surgery, would help clarify the temporal relationships among these connectivity features, disease progression, and functional recovery. Finally, medication use was not incorporated as covariates in the primary connectivity analysis. Therefore, residual confounding by clinical heterogeneity cannot be excluded. Future studies with larger cohorts should incorporate these variables as covariates.

## 5. Conclusions

The present study showed that resting-state MEG functional connectivity quantified using multiband wPLI features revealed disease-related cortical network alterations in CSM and supported explainable machine learning-based classification in the study sample. The most prominent discriminative information was derived from long-range theta-band connections. These findings provide preliminary evidence that CSM may be associated with measurable supraspinal network reorganization and support the feasibility of investigating MEG-derived connectivity features as candidate neurophysiological biomarkers. Larger multicenter studies with independent external validation are required to establish the reproducibility, generalizability, and potential clinical utility of these candidate biomarkers.

## Figures and Tables

**Table 1:** Demographic data and function scores.

	HC	CSM
Age	58.3 ± 7.5	58.0 ± 8.0
Sex (male/female)	15/17	14/17
Height (cm)	165.6 ± 8.4	168.4 ± 7.5
Weight (kg)	69.2 ± 10.1	73.1 ± 13.4
Duration of symptoms (months)	-	11.6 ± 13.0
VAS score	-	3.3 ± 1.6
JOA score	-	11.3 ± 1.7
SF-36 score		
PF, physical function	61.2 ± 19.6	50.3 ± 21.1
RP, role physical	69.0 ± 31.9	41.1 ± 33.9
BP, bodily pain	81.8 ± 17.9	58.7 ± 19.2
GH, general health	70.0 ± 15.0	54.0 ± 19.6
VT, vitality	72.0 ± 13.6	55.1 ± 18.4
SF, social function	73.5 ± 17.3	56.9 ± 27.7
RE, role emotional	66.6 ± 32.3	44.1 ± 37.9
MH, mental health	63.5 ± 13.1	54.3 ± 13.7

HC, Healthy Control; CSM: Cervical Spondylotic Myelopathy.

**Table 2:** Performance Metrics of the 7 Machine Learning Models in Predicting CSM.

Machine Learner	Accuracy	Sensitivity	Specificity	AUC-ROC	AUC-PR	F1-Score
LR	0.714	0.789	0.661	0.844	0.854	0.722
RF	0.732	0.760	0.733	0.780	0.812	0.728
NB	0.746	0.709	0.781	0.766	0.798	0.723
SVM	0.714	0.703	0.748	0.753	0.816	0.695
KNN	0.637	0.789	0.539	0.712	0.768	0.675
XGB	0.715	0.720	0.731	0.709	0.714	0.703
DT	0.681	0.657	0.693	0.702	0.703	0.670

ROC, receiver operating characteristic; AUC, area under the curve; PR, precision recall; LR, logistic regression; RF, random forest; NB, naive Bayes; SVM, support vector machine; KNN, k-nearest neighbors; XGB, extreme gradient boosting; DT, decision tree.

## Data Availability

The datasets of the current study are not publicly available due to patient privacy concerns but are available from the corresponding author on reasonable request.

## Supplementary Materials

The following supporting information can be downloaded at: https://www.mdpi.com/article/10.3390/bioengineering13090988/s1, Table S1: Sensor composition of the eight ROIs; Table S2: Hyperparameter search spaces used for feature selection and machine-learning model optimization.

## Abbreviations

The following abbreviations are used in this manuscript:

CSM	Cervical spondylotic myelopathy
wPLI	Weighted phase lag index
PLV	phase-locking value
ROI	Regions of interest
SHAP	SHapley Additive exPlanations
HC	Healthy control
JOA	Japanese Orthopaedic Association
VAS	Visual Analog Scale
SF-36	36-Item Short Form Health Survey
PF	physical functioning
RP	role physical
BP	bodily pain
GH	general health
VT	vitality
SF	social functioning
RE	role emotional
MH	mental health
MSR	magnetically shielded room
ICA	independent component analysis
LR	Logistic Regression
SVM	Support Vector Machine
NB	Naive Bayes
KNN	K-Nearest Neighbors
RF	Random Forest
DT	Decision Trees
XGB	XGBoost
AUC	Area under the curve
PSD	Power spectral density
FDR	False discovery rate
ROC	Receiver operating characteristic
