# Supplementary material for: Resting-State Magnetoencephalography Functional Connectivity in Cervical Spondylotic Myelopathy: An MEG Study with SHAP-Based Interpretation
Source: Bioengineering (Basel). 2026 Aug 27;13(9):988. doi: 10.3390/bioengineering13090988 (PMC13603905; doi:10.3390/bioengineering13090988)
Supplement: Supplementary file 1 [file bioengineering-13-00988-s001.zip › Supplementary Table S1.pdf]

Supplementary Table S1. Sensor composition of the eight ROIs.

ROI	Sensors	Total
Occipital	O1, PO3, PO7, O2, PO4, PO8, Oz, Iz, POz	9
Parietal	P1, P3, P5, P7, P9, P2, P4, P6, P8, P10, Pz	11
Central	C1, C3, C5, C2, C4, C6, Cz	7
Centroparietal	CP1, CP3, CP5, CP2, CP4, CP6, CPz	7
Frontal	F1, F3, F5, F7, F2, F4, F6, F8, Fz	9
Frontal Pole	Fp1, AF3, AF7, Fp2, AF4, AF8, Fpz, AFz	8
Temporal	T7, FT7, TP7, T8, FT8, TP8	6
Frontocentral	FC1, FC3, FC5, FC2, FC4, FC6, FCz	7
