# Supplementary material for: Resting-State Magnetoencephalography Functional Connectivity in Cervical Spondylotic Myelopathy: An MEG Study with SHAP-Based Interpretation
Source: Bioengineering (Basel). 2026 Aug 27;13(9):988. doi: 10.3390/bioengineering13090988 (PMC13603905; doi:10.3390/bioengineering13090988)
Supplement: Supplementary file 1 [file bioengineering-13-00988-s001.zip › Supplementary Table S2.pdf]

Supplementary Table S2. Hyperparameter search spaces used for feature selection and machine-learning model optimization.

Model	Hyperparameter	Candidate values
All models	Number of selected features (*k*)	10, 20, 30, 40
LR	C	0.01, 0.1, 1, 10, 100
SVM	C	0.1, 1, 10, 100
SVM	gamma	scale, auto, 0.01, 0.1, 1.0
RF	n estimators	400, 800
RF	max depth	None, 4, 8, 12
RF	min samples leaf	1, 2, 4
RF	min samples split	2, 5, 10
DT	max depth	None, 3, 5, 8, 12
DT	min samples leaf	1, 2, 4, 6
DT	min samples split	2, 5, 10
DT	criterion	gini, entropy, log loss
KNN	n neighbors	3, 5, 7, 9, 11, 15
KNN	weights	uniform, distance
KNN	p	1, 2
Gaussian NB	var smoothing	10^{-12}, 10^{-10}, 10^{-9}, 10^{-8}
XGBoost	max depth	3, 4, 5
XGBoost	n estimators	400, 800
XGBoost	learning rate	0.03, 0.05, 0.1
XGBoost	subsample	0.7, 0.8, 1.0
XGBoost	colsample bytree	0.7, 0.8, 1.0
